# SWATH-MS Quantitative Proteomic Analysis of Deer Antler from Two Regenerating and Mineralizing Sections

**DOI:** 10.3390/biology10070679

**Published:** 2021-07-17

**Authors:** María López-Pedrouso, José M. Lorenzo, Tomás Landete-Castillejos, Louis Chonco, Francisco Javier Pérez-Barbería, Andrés García, María-Pilar López-Garrido, Daniel Franco

**Affiliations:** 1 Department of Zoology, Genetics and Physical Anthropology, University of Santiago de Compostela, 15872 Santiago de Compostela, Spain; mariadolores.lopez@usc.es; 2 Centro Tecnológico de la Carne de Galicia, Rúa Galicia Nº 4, Parque Tecnológico de Galicia, San Cibrao das Viñas, 32900 Ourense, Spain; jmlorenzo@ceteca.net; 3 Área de Tecnología de los Alimentos, Facultad de Ciencias de Ourense, Universidad de Vigo, 32004 Ourense, Spain; 4 Instituto de Investigación en Recursos Cinegéticos (IREC) y Sec. Recursos Cinegéticos IDR, Universidad de Castilla-La Mancha (UCLM), 02071 Albacete, Spain; tomas.landete@uclm.es (T.L.-C.); louis.chonco@uclm.es (L.C.); fjavier.perez@uclm.es (F.J.P.-B.); andresjose.garcia@uclm.es (A.G.); 5 Laboratorio de Genética Médica, Instituto de Investigación en Discapacidades Neurológicas (IDINE), Facultad de Medicina, Universidad de Castilla-La Mancha (UCLM), 02071 Albacete, Spain; mariap.lopez@uclm.es

**Keywords:** deer antlers, bone metabolism, oxidative stress, heat shock proteins, glutathione, mass spectrometry, gene ontology, bioinformatic analysis

## Abstract

**Simple Summary:**

Deer antler is a unique and astonishing case of annual regeneration in mammalians. Several studies have pointed out the potential for use of velvet antler extract as a nutraceutical supplement, among others, because of its anti-cancer activity. The study of antler regeneration and growth allow us to identify the main proteins and regulatory pathways involved in cell differentiation and regeneration. For this purpose, two sections of antlers (tips and middle sections) using ribs as controls were analyzed from a proteomic point of view. A total of 259 proteins mainly associated with antioxidant mechanisms and Wnt signalling pathways could be responsible for deer antler regeneration and these proteins may be linked to human health benefits. Further studies should be focused on discovering which proteins from velvet antler extracts are associated with these beneficial effects.

**Abstract:**

Antlers are the only organ in the mammalian body that regenerates each year. They can reach growth rates of 1–3 cm/day in length and create more than 20 cm^2^/day of skin in the antler tips (their growth centers). Previous proteomic studies regarding antlers have focused on antler growth centers (tips) compared to the standard bone to detect the proteins involved in tissue growth. However, proteins of cell differentiation and regeneration will be more accurately detected considering more growing tissues. Thus, we set out to compare proteins expressed in antler tips (the highest metabolism rate and cell differentiation) vs. middle sections (moderate cell growth involving bone calcification), using ribs as controls. Samples were obtained in mid-June with antlers’ phenology corresponding to the middle of their growth period. Quantitative proteomic analysis identified 259 differentially abundant proteins mainly associated with antioxidant metabolic mechanisms, protein formation and Wnt signalling pathway, meanwhile, the mid antler section was linked to blood proteins. The high metabolic rate and subsequent risk of oxidative stress also seem to have resulted in strong antioxidant mechanisms. These results suggest that redox regulation of proteins is a key factor in the model of deer antler regeneration.

## 1. Introduction

Deer antlers are bony cranial appendages which are renewed each year. In the case of red deer, the antlers grow in about 3.5 months in red deer [1]. They constitute the only case of full regeneration in mammalian organisms, and they show an enormous growth rate in the tips, growing up to 1–3 cm/day in length, and creating more than 20 cm^2^/day of skin [2,3]. Indeed, a recent study has shown that antlers have evolved a speed of growth faster than cancer based on the high expression of proto-oncogenes [4]. As a result, the study postulated that several tumor genes (e.g., TP53) could be suppressed to control the high risk of developing cancer. In this sense, several studies have found in vitro and in vivo anti-cancer effects of deer antler velvet extract in human tumors such as glioblastoma [5], prostate [6,7], colon [8] and breast [9]. Velvet antler has been used as traditional medicine for over 2000 years and it is recognized in the pharmacopoeias of China, Korea and Japan. Furthermore, it has been claimed as a nutraceutical supplement in New Zealand, the USA and Canada [10]. In this sense, it has been reported that polypeptides and proteins are the main bioactive components of the deer antler velvet [11], suggesting that the anti-cancer activity of velvet antler extract is mainly due to their proteins or peptides. Furthermore, the peptide extracts from the antler growth center have protective effects against oxidative stress. Thus, some studies found that differentially expressed proteins are involved in the regulation of several pathways such as oxidative phosphorylation, ribosome or extracellular matrix interaction [12], whereas others found a tetrapeptide (YNVK) that exhibited strong antioxidant activity [13].

Recently, proteomic analysis has been used to examine which proteins may be responsible for the fast growth and regenerative capacity of velvet antler [11,12,14,15]. The regenerative capacity of the antlers comes from the pedicle periosteum (PP) and the resident cells. Antler regeneration involves a process from dormant to potentiated active state due to stem cells [14]. Additionally, plasma membrane proteins are of key importance to different cellular processes. The proteome of plasma membrane proteins of stem cells in the PP compared to control cells from the skull (facial periosteal cells) showed differences in external stimuli, signal transduction, membrane transport, regulation of tissue regeneration and protein modification processes [16]. Similarly, a quantitative proteomic analysis of antlerogenic periosteal cells compared with the facial periosteal cells from yearling deer was performed. Overexpressed extracellular proteins in antlerogenic periosteal cells were found and these proteins are involved in cell proliferation, angiogenesis and neurogenesis. The comparison of extracellular and intracellular proteomes suggested secreted proteins that might regulate antler formation and regeneration, such as SFRP4 and LUM [11]. Beyond this, two different antler systems harvested from red deer and sika deer were analyzed. More abundant proteins were found in sika deer than red deer and these proteins are involved in oxidative phosphorylation, ribosome, extracellular matrix interaction and the PI3K-Akt pathway. For a better understanding, several antler sections with different growth rates should be compared. Thus, the proteomes in several sections of the antler assessed stem cells under different stages of activation [17]: dormant pedicle periosteum at the base of the antler, the antler growth center in the tip and mid sections of the antler beam periosteum. The authors found the greatest number of unique proteins (87) in the growth center of antler tips which could be associated with the activation of antler stem cells.

Despite being a fast-growing tissue, studies about velvet antlers are rather scarce. Most proteomic and transcriptomic studies have examined the growth center (tip) compared with the antler base or even the bones of the skull. These studies compare two confounding factors: fast growth in the tip, but also a differentiating center, with antler base or skull bone which is not very active. This study aims to compare the proteome of two sections of the antler with different metabolism rates, using deer ribs as controls: the antler tip (growth center), which has a highest metabolism and differentiating tissue, and the middle section of the antler, which also has a high metabolism, but due to its intensely mineralizing section and differentiated bone.

## 2. Materials and Methods

### 2.1. Sample Collection of the Deer Antler

In this study, we examined deer antlers from wild individuals in a stage of growth phenotypically corresponding to the mid-growth period. Within each antler, we examined the proteome of the antler tip (growth center) as compared with that of the middle sections (corresponding to intensely mineralizing antler bone). As a control, we examined the ribs of the same individuals.

We used samples from six adult males, selected from a larger group that were hunted to reduce population density (for game management purposes) in a deer private game state in Ciudad Real (Spain) according to existing legal regulations. The slaughter of the hunted animals was regulated by the Regional Hunting Law of Castilla la Mancha [18]. Of the animals hunted, the six selected had antlers in the phenotypical aspect of being in the middle of their growth period (approximately 60 days of growth), based on antler velvet grading of Deer Industry New Zealand (https://www.pggwrightson.co.nz/-/media/Corporate/Documents/Velvet/Velvet-DINZ-Grading-Guidelines.pdf?la=en, accessed on 15 July 2021). Immediately after death, antlers were cut off using a mechanical saw and stored frozen at −20 °C. No blood or skin were removed because, depending on the protocol to extract the blood, different amounts could remain, increasing the variability of results. The antlers were sampled in two sections: (1) the tip (considered as the 2.5 cm top section of the main beam) and a section of 5 cm in the middle of the antler. The same process was conducted for middle sections of floating ribs of 5 cm. Each sample was lyophilized, homogenized by trituration in an ultracentrifuge mill (Retsch ZM-100) and the triturate was lyophilized again and stored in plastic containers at −20 °C for later analysis.

### 2.2. Protein Extraction and Tryptic Digestion

The deer antler powder (50 mg) was dissolved in RIPA buffer (200 mmol/L Tris/HCl-pH 7.4, 130 mmol/L NaCl, 10%-*v/v* glycerol, 0.1%-*v/v* SDS, 1%-*v/v* Triton X-100, 10 mmol/L MgCl_2_) and anti-proteases and anti-phosphatases (Sigma-Aldrich, St. Louis, MO, USA). A homogenization was prepared using TissueLyser II (Qiagen, Tokyo, Japan) and then centrifuged at 14,000× *g* (4 °C, 20 min). An RC-DC kit (Biorad Lab., Hercules, CA, USA) was employed to assess the protein concentration according to its instructions. A total amount of 100 μg was loaded on 10% SDS-PAGE to concentrate the proteins in a gel single band. Thus, the band was excised into pieces and washed with Milli-Q water, followed by 50 mM ammonium bicarbonate in 50% methanol. The dehydration of gel material was carried out with a vacuum centrifuge. Afterwards, the protein sample was reduced by 10 mM DTT and 50 mM ammonium bicarbonate solution at 60 °C for 30 min followed by the alkylation by 55 mM iodoacetamide and 50 mM ammonium bicarbonate solution at room temperature for 30 min in darkness. Finally, digestion by 20 ng/μL trypsin (Promega, Madison, WI, USA) in 20 mM ammonium bicarbonate incubating at 37 °C was used on the final solution. The resulting peptides were dissolved in 0.1% formic acid and stored at −20 °C for later analysis.

### 2.3. Protein Identification and Reference Spectral Library Building

A composite sample for the two groups was prepared to mix 4 μg of protein from each sample. The resulting solution was then assessed by analysis by shotgun data-dependent acquisition (DDA) employing micro-LC system Ekspert nLC425 (Eksigen, Dublin, CA, USA) using a YCM-TriartC18 column (150 mm × 0.3 mm i.d., 12 nm pore size, 3 μm particle size) (YMC CO., Kyoto, Japan). The solvents were: solvent A (water, 0.1% formic acid) and solvent B (ACN, 0.1% formic acid). The gradient consisted of 5–95% B for 30 min, 5 min at 90% B and finally other 5 min at 5% B for column equilibration, for a total time of 40 min using a flow rate of 5 μL/min. The detection was carried out by a hybrid quadrupole-TOF mass spectrometer, model Triple TOF 6600 (SCIEX, Framingham, MA, USA) operating with a data-dependent acquisition system in positive ion mode. The working parameters were 250 ms survey scan from 400 to 1250 *m/z* followed by MS/MS experiments from 100 to 1500 *m/z* (25 ms acquisition time) for a total cycle time of 2.8 s. The fragmented precursors were added to the dynamic exclusion list for 15 s, any ion with charge +1 was excluded from the MS/MS analysis. The comparison of mass spectral data and databases was performed by ProteinPilot software v.5.0.1. (SCIEX, Framingham, MA, USA). The used database was the Uniprot Swiss-Prot database for *Cervus elaphus hippelaphus* (European red deer) using a false discovery rate (FDR) below 0.01 for peptides and proteins and a confidence score above 99%.

### 2.4. Protein Quantification by SWATH-MS

SWATH-MS (sequential window acquisition of all theoretical mass spectra) acquisition was performed using the data-independent acquisition (DIA) method. Six samples of both groups of deer antler (tip and middle section) and six from ribs with two technical replicates were assessed. An amount of 4 μg of protein was analyzed by LC under the above conditions. Regarding the MS/MS analysis, an acquisition time of 50 ms in a total cycle time of 6.3 s was performed. A cycle consisted of the acquisition of 65 scans per SWATH window of variable width (1 *m/z* overlap) covering the 400–1250 *m/z* mass range. The spectral alignment and targeted data extraction were performed by PeakView v.2.2. (SCIEX, Framingham, MA, USA) matching the reference spectral library. The DIA files considered were an extraction window of 5 min (a width of 30 ppm) using the settings: ten peptides/protein, seven fragments/peptide, excluded shared and modified peptides and FDR below 0.01. The final quantification was measured by adding the quantitative outputs from the peaks for fragments.

Two comparisons were performed to identify differentially abundant proteins (DAPs) using paired Student’s t-test and considering only *p*-values above 0.05 and fold change of 1.5 as the cut-off: (1) A comparison between the tip and middle section of deer antler (fastest-growing section vs. mineralizing one) and; (2) middle section of the antler vs. ribs (fast mineralization vs. standard bone metabolism). To evaluate the relationship among the three locations, a factorial analysis of the common DAP (*p* < 0.05) in the three tissues was carried out. Principal component analysis (PCA) was used as the method for extraction, and it was performed on the correlation matrix. A varimax rotation was carried out to minimize the number of variables that influence each factor, and thus, to facilitate the interpretation and discussion of the results. A KMO value of 0.767 was obtained. XLSTAT 2018.5.52745 software (Addinsoft, NY, USA) was used.

Gene Ontology (GO) and KEGG (Kyoto Encyclopaedia of Genes and Genomes) analyses of differentially abundant proteins were performed using the enrichment analysis tool, FunRich (Functional Enrichment analysis tool, http://www.funrich.org, accessed on 15 May 2021) [19].

### 2.5. ELISA Procedure for IGF-1 and IFN-γ Determination

Deer antler velvet powder (1 g) was weighed and soaked with 10 mL distilled water. The liquid mixture was incubated at 4 °C overnight with continuous stirring and then centrifuged at 2700× *g* for 20 min. The supernatant was freeze-dried and dissolved into 2 mL phosphate buffered saline (PBS, Lonza BioWhittaker). Samples were frozen at −80 °C and to carry out any further assay, samples were thawed, passed through a 0.22 μm filter (PES membrane, MERCKMILLIPORE, Molsheim, France) and centrifuged at 5600× *g* for 3 min. According to the manufacturer’s recommendations, deer IGF-1 ELISA kit (Catalog Number. CSBE12644, CUSABIO) and deer IFN-γ (Catalog No: EK11988, SAB) were performed.

## 3. Results

### 3.1. Comparisons of Deer Proteomes Tip vs. Middle Antler, and Middle Antler vs. Ribs

**Figure 1:**
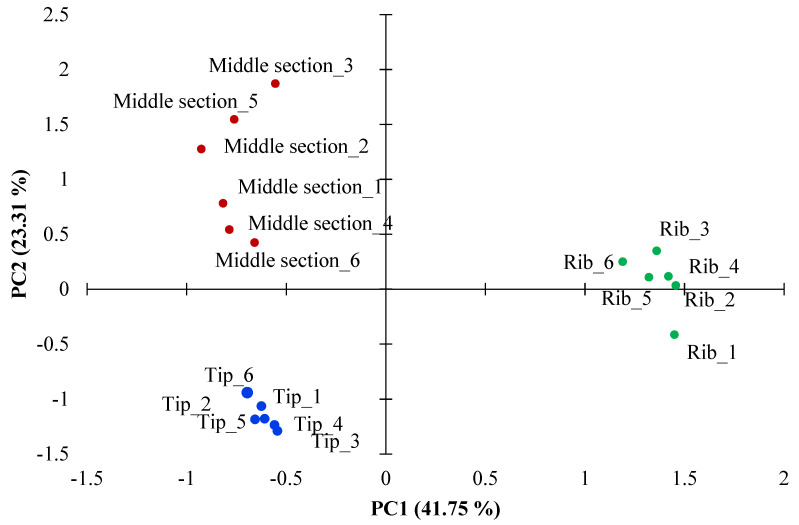
Projection of the antler samples of Iberian red deer (*Cervus elaphus*) of the three locations (tip, middle and rib) in the plane defined by the first two principal components.

In this study, 259 proteins were identified and quantified by SWATH-MS from two comparisons: tip and middle sections of deer antler velvet (Table 1) and, the middle antler section and the ribs (Table 2). The antler proteins from the tip and middle section and those from ribs were analyzed by PCA. According to PCA results, there were two components explaining 65.06% of the total variance. The two principal components can distinguish between the two sections of deer antler and the ribs. The first principal component (PC1) explained the higher percentage of variance (41.75%). The second principal component (PC2) accounted for 23.31% of the total variability indicated differences between middle section and tip of antler samples. The PCA figure shows that tip samples are more clustered, showing greater homogeneity than those that belong to the middle section resulting in more dispersal from the center of the cluster (Figure 1).

#### 3.1.1. Comparison Tip vs. Middle Antler

From the total proteins identified and quantified as DAP by SWATH-MS, those that exhibit a 1.5-fold between the two groups and a Student’s t-test with 5% statistical significance (*p* < 0.05) were considered as differentially abundant. In the comparison within the antler between the growth center (tip) and mineralizing section (middle), 34 proteins were overabundant in the tip (color blue) with 17 unidentified proteins (data not shown) and 44 proteins were overabundant in the middle section (color red) with 6 unidentified deer antler proteins (Table 1). The high number of unidentified proteins in both antler sections demonstrates the lack of knowledge of the proteomic profile of this tissue, especially in the tip section where 32.7% of the total DAPs were unidentified. In a previous study by our group on deer meat (*longisimus thoracis et lumborum*), the number of unidentified proteins, employing the same database, was lower [20], suggesting that the major constraint could be in the tissue of deer antler. This fact indicates that antler is not studied enough in terms of protein profile. In the middle section, the most abundant proteins (>10^5^) were hemoglobin subunit alpha, adult beta-globin 1, adult beta-globin 2, apolipoprotein A-II, alpha-1B-glycoprotein, serotransferrin, lactotransferrin and several unidentified proteins.

#### 3.1.2. Comparison Middle Section of Antler vs. Rib

In the comparison between the mineralizing section of the antler (middle section) and the ribs, 30 proteins were overabundant in the middle section (color red) with 6 unidentified proteins (data not shown), and 58 proteins were overabundant in the ribs (color green) with 26 unidentified deer antler proteins (data not shown) (Table 2). Among the majority of overexpressed proteins in the rib, the most abundant was the collagen alpha-2(I) chain (CO1Aa) with a fold change of 49, corresponding to gene COL1A2.

There is a set of proteins related to oxidative stress (chaperones, heat shock proteins and other types) overexpressed in the ribs compared to the mineralizing section of the antler. CAH3, glutathione S-transferase Mu 1, F10A1, protein disulfide-isomerase A4, SODC, glutathione S-transferase P, peroxiredoxin-6 and EF1G are the most relevant.

### 3.2. Comprehensive Analysis of Deer Antler Proteome

**Figure 2:**
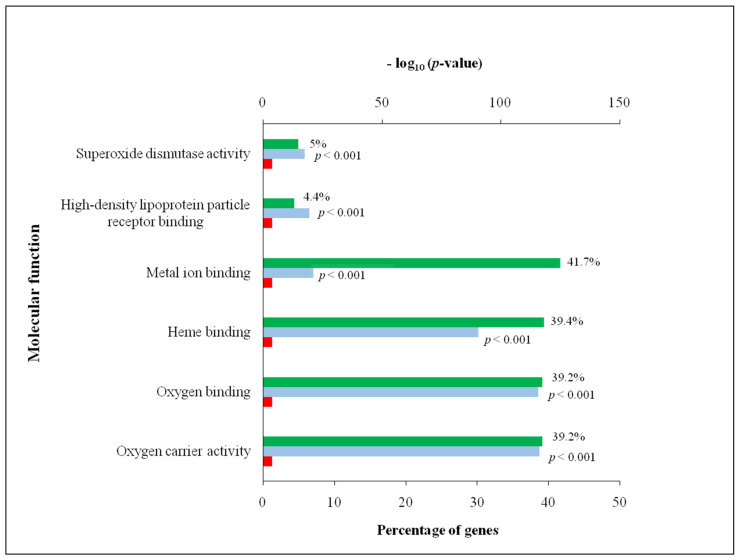

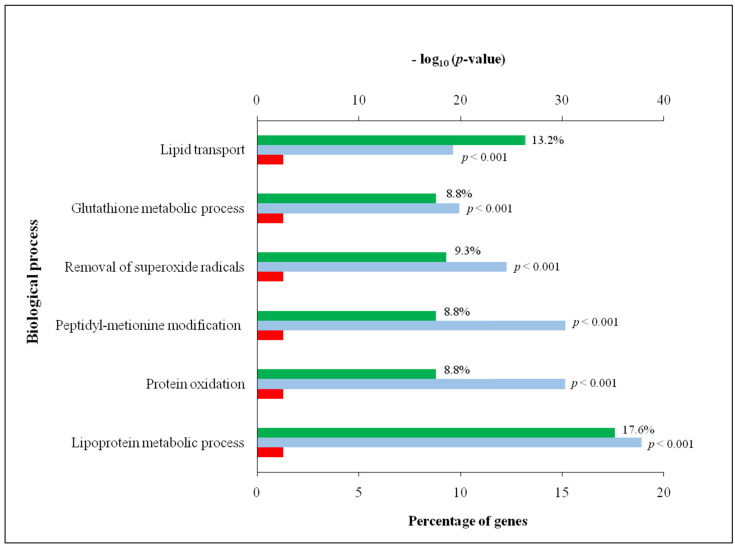
Functional enrichment analysis of differentially abundant proteins (expressed in percentage) of the antler samples of Iberian red deer (*Cervus elaphus*) of the two locations (tip and middle) using FunRich. The biological processes and molecular functions obtained were sorted by −log_10_ (*p*-value) and only the top 6 are displayed. The percentage of genes, *p* = 0.05 as reference and −log_10_ (*p*-value) are indicated in green, red and blue, respectively.

Deer antler contains several proteins with distinctive functions as can be concluded from Figure 2. The functional enrichment analysis for GO was performed using FunRich from DAPs between the tip and middle section. Biological process and molecular function were considered to carry out GO analysis of annotated proteins. Regarding biological processes, the more abundant categories were the lipoprotein metabolic process (GO:0042157), protein oxidation (GO:0018158), peptidyl methionine modification (GO:0018206), removal of superoxide radicals (GO:0019430), glutathione metabolic process (GO:0006749) and lipid transport (GO:0006869).

## 4. Discussion

### 4.1. Comparisons of Deer Proteomes Tip vs. Middle Antler, and Middle Antler vs. Ribs

The differences in the cluster mean distances of the samples between the middle and tip section showed by PCA analysis can be explained based on two reasons. Firstly, the tip growth center is a clearly defined unit, consisting of the five tissue layers: RM = reserve mesenchyme, PC = precartilage, TZ = transition zone, CA = cartilage and MC = mineralized cartilage [21,22]. The abundance of these proteins and gene expression were related to cartilage development and fast growth, and mineralization, together with those common to the organ’s development process [22]. On the other hand, the increase in the heterogeneity in the cluster of the samples from the middle section might be due to differences in the growth stage and the different sizes of the antlers.

#### 4.1.1. Comparison Tip vs. Middle Antler

The most predominant proteins identified in the middle section are related to blood physiology, which is not surprising as mineralizing antler has the highest proportion of filling osteons formed by blood vessels bringing proteins, minerals and glucose to supply the energy needed for bone formation. In the case of transferrins (serotransferrin and lactotransferrin), hemoglobin and adult beta-globin 1 and 2, they are related to iron ion binding. Blood physiological processes are also the least abundant but still overexpressed in the mineralizing section such as complement C3, heparin cofactor 2 (HEP2), hemopexin, antithrombin-III (ANT3), among others. Some of these proteins may be present because they play other roles. Such is the case of serotransferrins, which are responsible for iron transport from absorption sites and Fe-heme degradation to those of storage and utilization. The Fe^+2^ released into the bloodstream is oxidized to Fe^+3^ by plasma ferroxidases for their incorporation into serotransferrin. Thus, iron is distributed among cells and used as a cofactor in many enzymatic reactions. One of them, highly important in the tissues of high metabolic rate, is to form part of catalase, one of the main antioxidant enzymes in control of oxidative stress [23,24,25]. Moreover, this protein might also have a role in stimulating cell proliferation [26]. In the case of lactotransferrin, a glycoprotein that also can link to iron, several biological activities were also described: iron homeostasis, anti-microbial and immunomodulatory effects and anti-tumor activity [27].

Apolipoprotein A-II is the second most abundant apolipoprotein in the high-density lipoproteins (HDL), but its function is still unclear. However, its function could be related to afamin, which is overexpressed in the middle section. Little is known about the physiological function of afamin apart from the fact that is a carrier of vitamin E. A rather recent study has shown that afamin is co-expressed (thus, expected to have a similar DAP level) in a ratio of 1:1 with proteins of the Wnt signalling pathway, particularly Wnt-3 [28]. The same study showed a somewhat lower co-expression of apolipoprotein. The Wnt proteins consist of a large family of glycoproteins which mainly control embryonic development and adult homeostasis. The Wnt signalling pathway is related to embryonic or organ development, and therefore, its genes are also highly expressed in antler development [3,9,29]. The proteins of the Wnt pathway are lipoproteins, and afamin and apolipoprotein A-II seem to play a role in helping to dissolve and carrying lipoproteins highly important in antler development signalling by Wnt3 and other Wnt proteins. The apolipoprotein A-II and serotransferrin are related to the regulation of IGF transport and uptake by insulin-like growth factor binding proteins (IGFBPs), whose family of growth factors are involved in antler development [30].

Alpha-1B-glycoprotein, another DAP, is a glycoprotein from plasma whose function is not clear. Indeed, it was reported that it might be a novel member of the immunoglobulin superfamily involved in cell recognition/regulation [31]. In humans, this protein has been related to several diseases. For instance, bladder cancer has been identified in all tumor-bearing patient samples but not in samples obtained from non-tumor-bearing individuals [32].

Another of the main DAPs was alkaline phosphate with a fold change of 4.00 (Table 1). This is the most important protein for incorporating Ca^2+^ from the bloodstream into the mineralizing bone, and it is abundantly found in the antler mineralizing tissue until it is fully mature when it disappears [33].

In the case of deer antler tip, the most DAPs were endoplasmin, 60 S acidic ribosomal protein P0, Y-box-binding protein 1 and Hsc70-interacting protein. Among them, the endoplasmin and Hsc70-interacting proteins are molecular chaperones involved in unfolded protein binding. The overabundance of such proteins in the antler growth center could be associated with the fast-growing of this tissue synthesizing a large number of proteins. These molecular chaperones ensure folding of the newly synthesized proteins in the cell into their tridimensional structure. In this sense, 60 S acidic ribosomal protein P0 and Y-box-binding protein 1 are also related to protein formation, through ribosome activity [34]. Similar functions of protein synthesis seem to have other ribosomal proteins with high content in the antler tip, such as ribosome-associated molecular chaperone SSB1, 60 S acidic ribosomal protein P2 (RLA2), 60 S acidic ribosomal protein P1 (RLA1) or heterogeneous nuclear ribonucleoprotein K (HNRPK) [35].

**Figure 3:**
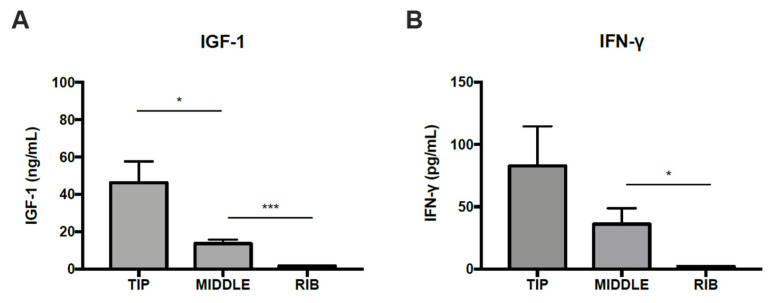
(**A**) Concentration of IGF-1 in two sections of antlers and internal bone (rib) as a control. (**B**) Concentration of IFN-γ in two sections of antlers and internal bone (rib) as a control. Data are means ± standard error of the mean. *p*-value of the significant comparison against control (* *p* = 0.05–0.01, *** *p* ≤ 0.001).

The DAPs with antioxidant capacity are particularly interesting. It would be expected that the expression of antioxidant proteins is increased in fast-growing tissues. Although both (antler and mineralizing part) have high metabolic rates, this seems to be higher in the tip, according to IGF-1 and IFN-γ levels (Figure 3). In the comparison of mineralizing antler vs. rib, a higher content of antioxidant molecules in the antler could be assumed. However, the results seem to be much more complex. The antioxidant proteins peroxiredoxin-2, carbonic anhydrase (CAH3), glutathione transferase, Cu/Zn superoxide dismutase (SODC), and peroxiredoxin-6 were more abundant in the mineralizing part of the antler, meanwhile, glutathione S-transferase P was more abundant in the tip.

#### 4.1.2. Comparison Middle Section of Antler vs. Rib

The collagen alpha-2(I) chain is an important part of the bone organic extracellular matrix [36], suggesting a high content in the proteome of mature and full-grown bone (rib) compared to the forming bone (mineralizing section) with their osteons still only partly filled. Surprisingly, the collagen alpha-1(I) chain corresponding to gene COL1A1 was more abundant in the mineralizing section of the antler (3 times more expressed in antler than in rib as shown in Table 2). Remarkably, both genes COL1A1 and COL1A2 were overexpressed by a factor of 10 or 20 in the mineralizing section of the antler as compared to the rib of the same animal [29,37]. The seemingly contradictory results indicate gene expression is related to the rate of protein production, whereas the proteome is related to the abundance of protein. Accordingly, the mature bone may have lower rates of gene expression COL1A2 in the rib. However, the bone organic matrix may have a much greater amount of its related protein, CO1Aa. In the case of COL1A1, despite the high overexpression of the gene, the amount of protein may be only 3 times greater than its content in mature bone. It would be part of the scaffold which osteons are filling [38], thus being proportionately greater in growing antlers than in fully mature bone.

One of the most surprising results was that the second most abundant protein (12-fold overexpression) in the deer ribs, that is the fatty acid-binding protein (FABP4), found in adipocytes with the function of fatty acid-binding, but also inflammatory response. Although it suggests that yellow bone marrow might be found in ribs, the more likely reason relates deer ribs with osteoporotic bones. The antler grows at such speed that the deer suffer an annual cycle of osteoporosis to deliver skeleton proteins and minerals to the growing antler [29,39,40]. Borsy et al. assessed [39] the gene expression of deer ribs (one of the most prominent bones suffering such osteoporosis) and bones of human patients searching for an osteoporosis model. Several new genes, including FABP4, were found in common between deer cyclic and human pathological osteoporosis. FABP4 is more expressed in both types of osteoporosis. In addition, several studies have found that FABP4 is present in mouse embryos in adipocyte-like cells in non-fat tissues, including cartilage primordia and vertebrae [41]. This fatty-acid binding protein may bind proteins of the Wnt signalling pathway in connection with lipoproteins [28]. In the case of the deer rib (compared to the middle antler section), the need to bind Wnt would be related to intense bone remodeling caused by annual osteoporosis. 

The set of antioxidant proteins (glutathione S-transferase Mu 1, F10A1, protein disulfide-isomerase A4, SODC, glutathione S-transferase P, Peroxiredoxin-6, EF1G) overexpressed in the ribs compared to the mineralizing section of the antler are linked to oxidative stress. This important biological process is connected to the production of ROS and strongly associated with high metabolic rate and the ability of the organism to counteract them [23]. Although deer ribs are in a stage of high bone resorption to support antler growth, known as cyclic osteoporosis [29], its metabolism rate should be lower than one of the mineralizing parts (middle section of antler) suggesting a greater content in ribs. Another protein that should be expected to be higher in mid parts of the antler than in ribs is cellular retinoic acid-binding protein 2 related to limb morphogenesis. Further studies on the cellular metabolic pathways in deer antler vs. osteoporotic bones are required to understand these complex interactions.

On the other hand, several proteins are overexpressed in the mineralizing parts of the antler as compared to ribs. Several are related to Ca^2+^ transport or fixation into the mineralizing antler, such as albumin (5-fold greater in mid antler), plasminogen (involved in tissue remodeling, similar overexpression), amiloride-sensitive amine oxidase, hemopexin (which also has functions in remodeling of blood vessels).

The most abundant proteins in the mineralizing antler are related to blood physiology, largely due to blood vessels occupying most of the filling osteons. Among these proteins, heparin cofactor 2 (HEP2), complement C3, antithrombin-III (ANT3), hemoglobin subunit alpha, apolipoprotein A-I, hemopexin, fibrinogen alpha chain and heparin cofactor 2 (HEP2) were found. A few of them have other important functions that are expected in the tissue of high metabolic rate, such as the antler. In this sense, creatine kinase B-type is involved in binding ATP, probably from the bloodstream into velvet antler cells, and serotransferrin (TRFE) is linked to cell proliferation. Particularly interesting functions among DAP proteins in the tip vs. mid sections may relate to the anti-cancer activity mentioned earlier; recently, Chonco et al. [5] reported properties against glioblastoma in an extract from the tip, but not from the middle parts of the antler. However, it remains to be elucidated which protein or peptide is responsible for this important activity.

### 4.2. Comprehensive Analysis of Deer Antler Proteome

The lipoprotein metabolic processes shown by GO analysis may indeed point to the fact that antler growth is mostly related to the Wnt signalling pathway [3,28,29,37]. These functions are included in the protein metabolic process. Indeed, the skeletal growth factor is closely related to the bone matrix that stimulates proliferation and protein synthesis in bone cells. The regulation of bone formation depends on several growth factors such as IGF-1 and others as previously mentioned [42]. Protein oxidation and peptidyl methionine modification are also connected to the cellular protein modification process. In the case of protein oxidation, it has been reported that the reduction-oxidation stability is a key step in the modulation of bone cell function and the mineralization of tissues. This balance could be modified to change the activity in bone cells in the treatment of bone diseases [43]. Regarding the biological process, the lipid transport of the present study resulted in significant (*p* < 0.001) differences between the tip and the middle section (Figure 2). In addition to the mentioned role played by lipoproteins of the Wnt family in antler growth, these proteins may also be related to energy metabolism which is crucial for such a fast-growing tissue. Thus, there is a connection between fatty acid and glucose metabolism, contributing to energy homeostasis via lipoprotein lipase which plays a key role as a regulator of fatty acid transport through skeletal compartments [44].

Oxygen is essential for living organisms to address their energy metabolism needs but the presence of oxygen leads to ROS production. The inability of cells and organisms to counteract ROS during cellular metabolism leads to oxidative stress, particularly in the mitochondria, and a cycle of DNA damage, further impairment of ROS counteracting, greater oxidative stress and further cell damage occurs [23]. Indeed, the cellular redox imbalance were studied in connection with apoptosis, ageing and several pathological conditions, particularly cancer [23,45]. To restore homeostasis, organisms have a potent battery of cell endogenous antioxidants, e.g., superoxide dismutase (SOD), catalase (CAT) and glutathione peroxidase (GPX) that trigger a cascade of biological processes to block these free radicals. Indeed, glutathione helps to support antioxidant defense in cellular events such as DNA and protein synthesis, cell proliferation, signal transduction and immune response relevant to cancer diseases, heart attack, diabetes and other diseases [46]. The ROS activate the response of the antioxidant endogenous system acting synergically. For instance, SOD scavenging activity is increased in parallel with GPX and CAT. Indeed, the main function of SOD is to convert superoxide to hydrogen peroxide removed by GPX or CAT, meanwhile, the glutathione system is activated to protect protein thiols. In the present study, the pathways involving glutathione and SOD were altered from the middle section to the tip of the deer antler (Table 1; Figure 2). SOD activity certainly resulted in a significant difference in the middle section than the tip (FC = 1.85; Table 1). Concerning glutathione metabolism, two isoforms of GSH (glutathione transferase and glutathione S-transferase P) were significantly more abundant in each section. One isoform was more abundant in the middle section (FC = 3.12; Table 1), whereas the other isoform was more abundant in the tip section (FC= 0.55; Table 1). Dysfunctions of chondrocytes, as the unique cell in mature cartilage, are associated with mitochondria dysfunctions causing a redox imbalance [47].

On the other hand, heat shock proteins and other chaperones involved in protein folding and aggregation, as well as translocation reactions, are considered stress proteins. The rapid growth and reorganization of tissues of the deer antler could be largely due to these proteins. As can be observed in Table 1, the heat shock 70 kDa protein 5 was significantly more abundant in the tip of deer antler (FC = 0.55). This family of proteins is extensively studied and it has been reported that heat shock 70 kDa modulates numerous factors such as the cycle of cell division, growth factors, cell differentiation, tissue development or hormonal stimulation caused by stress conditions, among others [48].

Finally, the array of proteins related to blood metabolism and physiology in the middle part of the antler indicates that the mineralizing part of the antler is richly vascularized, as well as the importance of blood supply to bring metabolites needed for fast mineralization and tissue maturation. As shown in Figure 3, the highest concentrations of IGF-1 and IFN-γ in the tip of the deer antler in comparison to mid parts and the ribs confirmed that deer antler velvet has many active components involved in cell regeneration and inflammatory reaction. Hence, this finding has shown that the presence of IGF-1 and IFN-γ in each antler section compared to the ribs, is an example of the velvet antler active components contained in the velvet antler such as glycosaminoglycans, phospholipids, hormones, and growth factors (IGF-1, nerve growth factor and epidermal growth factor) as indicated by Chunjuan [49].

## 5. Conclusions

Major differences in the deer antler proteome were found between the tip and the middle section, and between the latter and ribs. In this regard, 76 proteins were detected as differentially abundant proteins for the first comparison, and 85 for the second. Those proteins define the differential properties of these two tissue locations in the velvet antler. Distinctive functions of these proteins were allocated from the functional enrichment analysis, highlighting the relevance of the oxidative stress among them. Indeed, protein oxidation, removal of superoxide radicals and glutathione metabolic process resulted in great distinction between the two sections. Additionally, alterations of proteins of redox regulation directly affected energetic and metabolic pathways suggesting a key role in this model of deer antler regeneration. Further research needs to be done in greater detail to understand the development of this tissue.

## Figures and Tables

**Table 1:** List of differential abundant proteins (mean value ± standard error of mean) of the antler samples of red deer (*Cervus elaphus*) from a native Iberian population of the two locations (tip and middle), gene names and fold change (FC) between both locations.

Items	Protein Name	Gene Names	Middle Section	Tip	FC
			Mean ± SE	Mean ± SE	
	Hemoglobin subunit alpha	HBA/SCN2A	2,563,475.0 ± 869,235.6	155,785.3 ± 39,216.0	16.46
	Adult beta-globin 1	HBB	45,176,500.0 ± 16,757,107.7	3,050,606.7 ± 1,088,464.0	14.81
	Adult beta-globin 2	HBB	13,515,916.7 ± 4,092,463.0	1,083,311.7 ± 331,202.8	12.48
	Creatine kinase B-type	CKB	311,250.0 ± 37,004.7	40,426.8 ± 3039.0	7.70
	Alpha-2-macroglobulin (A2MG)	A2M	316,528.3 ± 77,031.0	52,790.2 ± 5947.1	6.00
	Inter-alpha-trypsin inhibitor heavy chain H1	ITIH1	165,838.3 ± 53,177.3	30,431.5 ± 1875.7	5.45
	Amiloride-sensitive amine oxidase	AOC1	39,327.2 ± 6441.8	9712.9 ± 1135.1	4.05
	Carbonic anhydrase (CAH3)	CA3	22,310.6 ± 5010.6	5560.7 ± 1178.0	4.01
	Alkaline phosphatase (PPBN)	ALPG	15,993.0 ± 1618.5	4000.0 ± 467.9	4.00
	Inter-alpha-trypsin inhibitor heavy chain H2	ITIH2	87,390.0 ± 17,580.9	24,580.3 ± 2302.8	3.56
	Peroxiredoxin-2	PRDX2	37,583.5 ± 4204.3	11,079.8 ± 510.7	3.39
	Apolipoprotein A-II	APOA2	60,995.7 ± 16,710.0	18,302.5 ± 1743.9	3.33
	Alpha-amylase 1A	AMY1A	22,315.3 ± 4160.5	6740.0 ± 955.1	3.31
	Plasminogen (PLMN)	PLG	37,045.7 ± 9978.9	11,666.0 ± 644.5	3.18
	Glutathione S-transferase Mu 1	GSTM1	94,826.5 ± 25,325.8	30,432.3 ± 2409.7	3.12
	Complement C3	C3	712,043.3 ± 109,393.3	252,156.7 ± 27,537.1	2.82
	Afamin (AFAM)	AFM	25,198.7 ± 5103.3	9541.6 ± 1423.6	2.64
	Serpin 3-6	Serpin	378,931.7 ± 87,373.6	145,784.2 ± 24,405.9	2.60
	Mimecan (MIME)	OGN	55,036.5 ± 9328.6	21,511.8 ± 2205.4	2.56
	Alpha-1B-glycoprotein	A1BG	2,213,076.7 ± 441,457.5	866,086.7 ± 63,588.4	2.56
	Fetuin-B (FETUB)	Fetuin-B	56,124.7 ± 8673.8	22,155.8 ± 2673.3	2.53
	Alpha-2-antiplasmin (A2AP)	SERPINF2	24,572.3 ± 4954.9	9877.7 ± 579.0	2.49
	Complement factor B (CFAB)	CFB	90,554.5 ± 16,717.9	36,896.7 ± 2915.5	2.45
	Heparin cofactor 2 (HEP2)	SERPIND1	19,863.8 ± 3310.1	8067.4 ± 843.7	2.46
	Retinol-binding protein 4 (RET4)	RBP4	20,127.3 ± 4240.2	8274.5 ± 783.4	2.43
	Annexin A2	ANXA2	52,405.0 ± 7452.6	22,504.7 ± 1287.2	2.33
	Beta-N-acetylhexosaminidase	HEXB	4104.9 ± 607.5	1851.1 ± 250.1	2.22
	Lactotransferrin	LTF	1444,530.0 ± 231,686.6	659,118.3 ± 34,072.7	2.19
	Hemopexin (fragment)	HPX	173,172.2 ± 38,801.3	80,237.7 ± 9734.1	2.16
	Antithrombin-III (ANT3)	SERPINC1	64,056.0 ± 10,572.3	30,005.0 ± 1717.2	2.13
	40S ribosomal protein S27a (RS27A)	RPS27A	78,794.3 ± 10,336.1	38,137.8 ± 4586.8	2.07
	Apolipoprotein A-I	APOA1	4,144,766.7 ± 798,136.1	2,013,133.3 ± 167,607.1	2.06
	Alpha-1-acid glycoprotein 1 (A1AG1)	ORM1	95,798.0 ± 18,143.9	47,375.3 ± 3783.3	2.02
	Hemopexin (HEMO)	HPX	346,240.0 ± 72,507.6	172,841.7 ± 15,826.5	2.00
	Actin-depolymerizing factor	GSN	78,246.3 ± 13,025.7	39,946.8 ± 1462.5	1.96
	Serotransferrin (TRFE)	TF	2,125,016.7 ± 347,685.8	1,096,858.3 ± 76,391.0	1.94
	Adenosylhomocysteinase (SAHH)	AHCY	9665.6 ± 765.9	5004.9 ± 1092.1	1.93
	Lumican	LUM	206,390.0 ± 30,250.1	109,842.0 ± 11,393.3	1.88
	ATP synthase subunit beta (ATPB)	ATP5F1B	163,660.0 ± 11,615.0	87,713.2 ± 3889.4	1.87
	Cu/Zn superoxide dismutase (SODC)	SOD1	69,312.8 ± 7322.5	37,554.7 ± 3418.6	1.85
	Peroxiredoxin-6	PRDX6	88,053.3 ± 12,976.1	47,894.5 ± 2267.1	1.84
	Cystatin-B (CSTB)	Cystatin-B	26,072.0 ± 4123.7	14,697.6 ± 1446.9	1.77
	Transaldolase (TALDO)	TALDO1	12,790.6 ± 1895.0	7283.0 ± 1154.7	1.76
	Plastin-3	PLS3	32,357.3 ± 3587.1	18,817.3 ± 869.4	1.72
	Pyruvate kinase (KPYM)	PKM	171,291.7 ± 19,236.7	264,250.0 ± 9462.0	0.65
	L-lactate dehydrogenase	LDHB	100,577.0 ± 9101.6	155,815.0 ± 8110.5	0.65
	Polyadenylate-binding protein (PABP2)	PABPN1	5875.5 ± 1150.7	9190.6 ± 365.0	0.64
	Chloride intracellular channel protein 1	CLIC1	9348.2 ± 1694.4	14,520.7 ± 453.0	0.64
	Heat shock 70kDa protein 5	HSPA5	186,508.3 ± 33,958.7	301,630.0 ± 5751.1	0.62
	Heterogeneous nuclear ribonucleoprotein M	HNRNPM	10,044.3 ± 1370.7	16,531.0 ± 530.7	0.61
	Eukaryotic translation initiation factor 5A (IF5A1)	EIF5A	19,182.3 ± 3339.0	32,350.0 ± 3133.9	0.59
	Aggrecan core protein (PGCA)	ACAN	49,556.0 ± 4794.4	85,088.7 ± 5985.1	0.58
	Glutathione S-transferase P	GSTP1	24,142.5 ± 2739.0	44,152.7 ± 8435.2	0.55
	Peptidylprolyl isomerase	PPWD1	3916.0 ± 800.8	7129.1 ± 562.9	0.55
	Proteasome endopeptidase complex/PSB	PSMB	2137.7 ± 470.8	3933.2 ± 136.7	0.54
	Olfactomedin-like protein 3 (OLFL3)	OLFML3	9038.2 ± 1023.4	17,887.7 ± 2661.1	0.51
	Putative ATP-dependent RNA helicase	DHX57	3944.2 ± 1136.7	7865.7 ± 679.4	0.50
	Protein disulfide-isomerase	PDIA6	178,383.3 ± 22,517.8	390,683.3 ± 12,154.9	0.46
	Calponin	CNN2	3543.7 ± 896.7	8039.1 ± 1697.6	0.44
	Septin-7 (SEPT7)	SEPTIN7	3874.0 ± 1053.9	9256.4 ± 506.5	0.42
	Thrombospondin-1 (TSP1)	THBS1	8282.2 ± 2059.1	20,655.8 ± 2272.9	0.40
	Heterogeneous nuclear ribonucleoprotein (HNRPK)	HNRNPK	9548.3 ± 1725.5	24,761.2 ± 969.2	0.39
	Collagen alpha-1(II) chain (CO2A1)	COL2A1	26,222.3 ± 7333.0	67,945.7 ± 7406.7	0.39
	Glucosidase 2 subunit beta (GLU2B)	PRKCSH	2971.2 ± 894.4	8639.6 ± 737.5	0.34
	Protein disulfide-isomerase (fragment)	PDIA6	105,751.3 ± 16,292.9	334,988.3 ± 8084.2	0.32
	Heterogeneous nuclear ribonucleoproteins C1/C2	HNRNPC	1700.7 ± 405.7	6226.1 ± 350.6	0.27
	Hyaluronan and proteoglycan link protein 1 (HPLN1)	HAPLN1	63,678.3 ± 6588.1	235,995.0 ± 14,162.8	0.27
	Elongation factor 1-gamma (EF1G)	EEF1G	6767.1 ± 1803.8	25,447.8 ± 1352.1	0.27
	60S acidic ribosomal protein P1 (RLA1)	RPLP1	3699.7 ± 955.8	15,261.1 ± 1570.3	0.24
	60S acidic ribosomal protein P2 (RLA2)	RPLP2	8009.4 ± 1102.0	35,171.8 ± 3258.9	0.23
	Ribosome-associated molecular chaperone SSB1	SSB1	2193.3 ± 778.7	10,186.9 ± 346.4	0.22
	Elongation factor 1-delta (EF1D)	EEF1D	6809.5 ± 1064.5	32,352.3 ± 928.9	0.21
	Protein disulfide-isomerase A4	PDIA4	15,204.7 ± 2296.0	77,964.3 ± 3747.7	0.20
	Peptidyl-prolyl cis-trans isomerase (PIN4)	PIN4	25,527.3 ± 9262.3	132,480.8 ± 21,660.1	0.19
	Hsc70-interacting protein (F10A1)	ST13	2395.8 ± 725.3	14,398.3 ± 804.9	0.17
	Y-box-binding protein 1 (YBOX1)	YBX1	1189.9 ± 396.4	7032.0 ± 683.7	0.17
	60S acidic ribosomal protein P0 (RLA0)	RPLP0	1920.2 ± 265.3	12,717.7 ± 779.5	0.15
	Endoplasmin (ENPL)	HSP90B1	27,822.7 ± 4283.1	243,358.3 ± 8040.5	0.11

Proteins overexpressed in middle section and tip of antler are indicated in the table by red and blue color, respectively.

**Table 2:** List of differential abundant proteins (mean value ± standard error of mean) of the antler samples (middle section) of red deer (*Cervus elaphus*) from a native Iberian population compared to rib, gene names and fold change (FC) between both locations.

	Protein Name	Gene Names	Rib	Middle Section	
			Mean ± SE	Mean ± SE	FC
	Collagen alpha-2(I) chain (CO1Aa)	COL1A2	23,664,457.4 ± 4,097,670.2	481,201.8 ± 110,505.6	49.17
	Fatty acid-binding protein, adipocyte	FABP4	477,239.0 ± 58,119.2	40,306.7 ± 5080.5	11.84
	40S ribosomal protein S12		29,511.2 ± 7966.9	2719.0 ± 682.7	10.85
	Keratin, type II cytoskeletal 6A	KRT6A	340,515.4 ± 68,869.3	53,010.4 ± 16,401.4	6.42
	Fructose-bisphosphate aldolase	ALDOA	515,078.4 ± 93,413.7	81,491.0 ± 7750.3	6.32
	Carbonic anhydrase (CAH3)	CA3	128,113.8 ± 21,821.4	22,310.6 ± 5010.6	5.74
	Cellular retinoic acid-binding protein 2	CRABP2	14,697.1 ± 3304.0	2776.5 ± 616.7	5.29
	Hsc70-interacting protein (F10A1)	ST13	12,672.4 ± 1992.2	2395.8 ± 725.3	5.28
	Keratin, type I cytoskeletal 10	KRT10	360,962.0 ± 76,985.9	70,201.7 ± 19,902.1	5.14
	Transaldolase (TALDO)	TALDO1	60,475.7 ± 6149.7	12,790.6 ± 1895.0	4.72
	Y-box-binding protein 1 (YBOX1)	YBX1	5441.0 ± 1179.4	1189.9 ± 396.4	4.57
	Annexin A2 (fragment)	ANXA2	5372.1 ± 764.6	1182.3 ± 309.8	4.54
	Heterogeneous nuclear ribonucleoproteins C1/C2	HNRNPC	7177.4 ± 1158.7	1700.7 ± 405.7	4.22
	Alpha-amylase 1A	AMY1A	92,107.7 ± 12,024.1	22,315.3 ± 4160.5	4.13
	Protein disulfide-isomerase A4	PDIA4	61,477.6 ± 5982.7	15,204.7 ± 2296.0	4.04
	40S ribosomal protein S27a (RS27A)	RPS27A	308,538.6 ± 23,001.6	78,794.3 ± 10,336.1	3.92
	Glutathione S-transferase Mu 1	GSTM1	349,396.0 ± 39,083.3	94,826.5 ± 25,325.8	3.68
	Glucose-6-phosphate isomerase	GPI	56,018.1 ± 7541.4	15,638.1 ± 1216.3	3.58
	Heterogeneous nuclear ribonucleoprotein K(HNRPK)	HNRNPK	33,963.0 ± 5938.7	9548.3 ± 1725.5	3.56
	L-lactate dehydrogenase	LDHB	339,487.1 ± 68,674.4	100,577.0 ± 9101.6	3.38
	Polyadenylate-binding protein (PABP2) (fragment)	PABPN1	14,151.0 ± 3025.1	4320.8 ± 740.8	3.28
	Olfactomedin-like protein 3 (OLFL3)	OLFML3	28,187.8 ± 2177.8	9038.2 ± 1023.4	3.12
	Polypyrimidine tract-binding protein 1	PTBP1	12,884.6 ± 1449.8	4236.4 ± 382.5	3.04
	Putative ATP-dependent RNA helicase	DHX57	11,623.6 ± 2252.4	3944.2 ± 1136.7	2.95
	Tetranectin	CLEC3B	31,409.1 ± 5411.8	10,775.4 ± 2341.5	2.91
	60S acidic ribosomal protein P0 (RLA0)	RPLP0	5587.1 ± 730.0	1920.2 ± 265.3	2.91
	Triosephosphate isomerase	TPI1	548,662.6 ± 54,009.9	192,349.8 ± 28.673,1	2.85
	Ubiquitin-conjugating enzyme E2 variant 2	UBE2V2	14,901.8 ± 2303.7	5305.7 ± 1703.4	2.81
	6-phosphogluconate dehydrogenase decarboxylating	PGD	30,659.1 ± 3472.5	10,972.5 ± 2533.0	2.79
	Lupus La protein	SSB	6042.3 ± 964.5	2193.3 ± 781.8	2.75
	UDP-glucose 6-dehydrogenase	UGDH	8707.4 ± 1631.5	3215.2 ± 674.1	2.71
	Malate dehydrogenase, cytoplasmic	MDH1	11,937.7 ± 2189.3	4467.4 ± 1815.3	2.67
	Decorin	DCN	203,457.1 ± 19,766.6	78,507.4 ± 9628.3	2.59
	Phosphoglycerate kinase 1	PGK1	102,757.0 ± 15,094.9	39,944.3 ± 3442.8	2.57
	Peroxiredoxin-6	PRDX6	226,238.3 ± 16,359.3	88,053.3 ± 12,976.1	2.57
	Cu/Zn superoxide dismutase (SODC)	SOD1	173,733.6 ± 23,314.9	69,312.8 ± 7322.5	2.51
	Biglycan	BGN	100,093.6 ± 13,826.5	41,794.6 ± 5319,2	2.39
	Elongation factor 1-gamma (EF1G)	EEF1G	15,255.3 ± 2163.9	6767.1 ± 1803.8	2.25
	Glutathione S-transferase P	GSTP1	54,208.3 ± 5030.5	24,142.5 ± 2739.0	2.25
	Glucosidase 2 subunit beta (GLU2B)	PRKCSH	6611.3 ± 939.7	2971.2 ± 894.4	2.23
	Chloride intracellular channel protein 1	CLIC1	20,704.72 ± 3379.9	9348.2 ± 1694.4	2.21
	Glutathione S-transferase P (fragment)	GSTP1	42,414.8 ± 7016.7	19,194.5 ± 5.535.9	2.21
	60S acidic ribosomal protein P2 (RLA2)	RPLP2	17,289.1 ± 1634.2	8009.4 ± 1102.0	2.16
	Alpha-2-HS-glycoprotein	AHSG	274,143.4 ± 36,049.6	129,638.6 ± 29,231.7	2.11
	Transgelin	TAGLN	84,419.6 ± 10,442.4	40,987.1 ± 5652.8	2.06
	Transgelin (fragment)	TAGLN	37,823.7 ± 3877.1	18,390.6 ± 2628.0	2.06
	Calponin	CNN2	7285.9 ± 849.1	3543.7 ± 896.7	2.06
	Polyadenylate-binding protein (PABP2)	PABPN1	11,923.6 ± 887.0	5875.5 ± 1150.7	2.03
	Phosphatidylethanolamine-binding protein 1	PEBP1	17,399.2 ± 2682.0	8613.4 ± 1756.9	2.02
	Fatty acid-binding protein 5	FABP5	7163.9 ± 843.8	3625.2 ± 699.1	1.98
	Heterogeneous nuclear ribonucleoprotein H	HNRNPH1	31,298.3 ± 2793.2	16,102.7 ± 1632.7	1.94
	Endoplasmin (ENPL)	HSP90B1	51,370.1 ± 3589.4	27,822.7 ± 4283.1	1.85
	Lumican	LUM	380,040.3 ± 43,044.1	206,390.0 ± 30,250.1	1.84
	Transitional endoplasmic reticulum ATPase	VCP	68,467.5 ± 8692.5	39,195.5 ± 4453.9	1.75
	Phosphatidylethanolamine-binding protein 1	PEBP1	42,253.9 ± 5040.5	24,291.3 ± 4633.2	1.74
	Peptidyl-prolyl cis-trans isomerase (PIN4)	PIN4	375,942.8 ± 29,644.9	25,527.3 ± 9262.3	1.61
	UMP-CMP kinase	CMPK1	6599.5 ± 647.2	4105.2 ± 500.7	1.61
	Pyruvate kinase (KPYM)	PKM	261,337.6 ± 27,813.7	171,291.7 ± 19,236.7	1.53
	Galectin 1	LGALS1	49,839.4 ± 5621.5	76,707.49 ± 10,793.0	0.65
	Nucleobindin-1	NUCB1	24,434.2 ± 3012.5	38,920.5 ± 4924.8	0.63
	Heparin cofactor 2 (HEP2)	SERPIND1	11,873.5 ± 1969.7	19,863.8 ± 3310.1	0.60
	Elongation factor 2	EEF2	51,547.7 ± 4948.4	86,627.3 ± 12,626.1	0.60
	Plastin-3	PLS3	18,711.7 ± 2677.2	32,357.3 ± 3587.1	0.58
	Heat shock protein 70 1A	HSPA1A	65,543.3 ± 8657.8	114,598.05 ± 8998.6	0.57
	Serotransferrin (TRFE)	TF	1,215,178.8 ± 193,911.2	2,125,016.7 ± 347,685.8	0.57
	Inter-alpha-trypsin inhibitor heavy chain H2	ITIH2	47,937.9 ± 5216.0	87,390.0 ± 17,580.9	0.55
	Apolipoprotein A-IV	APOA4	45,231.5 ± 4775.7	85,435.1 ± 9548.5	0.53
	Nucleoside diphosphate kinase mitochondrial	NME4	67,760.2 ± 5520.7	129,968.3 ± 14,280.8	0.52
	Fibrinogen alpha chain	FGA	135,860.8 ± 21,986.1	291,686.2 ± 55,713.2	0.47
	Vimentin	VIM	156,778.7 ± 17,997.7	341,121.7 ± 67,935.6	0.46
	Hemopexin	HPX	79,373.2 ± 11,927.3	173,172.2 ± 38,801.3	0.46
	Afamin (AFAM)	AFM	10,596.6 ± 1972.3	25,198.7 ± 5103.3	0.42
	Alpha-1B-glycoprotein	A1BG	878,594.0 ± 101,549.5	2,213,076.7 ± 441,457.5	0.40
	Thioredoxin domain-containing protein 5	TXNDC5	47,903.8 ± 6878.6	121,210.10 ± 10,595.2	0.40
	Eukaryotic translation initiation factor 5A (IF5A1)	EIF5A	7520.5 ± 1315.2	19,182.3 ± 3339.0	0.39
	Transthyretin	TTR	98,470.6 ± 19,719.4	251,207.1 ± 73,458.0	0.39
	Apolipoprotein A-I	APOA1	1,599,381.1 ± 272,199.7	4,144,766.7 ± 798,136.1	0.39
	Creatine kinase B-type	CKB	119,883.7 ± 15,308.5	311,250.0 ± 37,004.7	0.39
	Amiloride-sensitive amine oxidase	AOC1	14,519.1 ± 3374.7	39,327.2 ± 6441.8	0.37
	Hemoglobin subunit alpha	HBA/SCN2A	905,083.4 ± 296,103.9	2,563,475.0 ± 869.235.6	0.35
	Antithrombin-III (ANT3)	SERPINC1	22,535.4 ± 2287.5	64,056.0 ± 10,572.3	0.35
	Apolipoprotein A-II	APOA2	20,745.2 ± 5114.2	60,995.7 ± 16,710.0	0.34
	Collagen alpha-1(I) chain	COL1A1	87,968.2 ± 11,426.9	275,756.5 ± 54,450.2	0.32
	Complement factor B (CFAB)	CFB	27,628.1 ± 3354.1	90,554.5 ± 16,717.9	0.31
	Histidine-rich glycoprotein	HRG	46,388.9 ± 4259.7	159,018.1 ± 45,430.2	0.29
	Albumin	ALB	4,096,318.3 ± 713,885.6	19,510,270.2 ± 3,628,081.0	0.21
	Plasminogen (PLMN)	PLG	7232.5 ± 2021.8	37,045.7 ± 9978.9	0.20
	Complement C3	C3	131,933.9 ± 14,398.5	712,043.3 ± 109,393.3	0.19

Proteins overexpressed in rib and middle section of antler are indicated in the table by green and red color, respectively.

## Data Availability

Not applicable.
